# A Hypothesis Test for Equality of Bayesian Network Models

**DOI:** 10.1155/2010/947564

**Published:** 2010-08-09

**Authors:** Anthony Almudevar

**Affiliations:** 1 Department of Computational Biology, University of Rochester, 601 Elmwood Avenue, Rochester, NY 14642, USA

## Abstract

Bayesian network models are commonly used to model gene expression data. Some applications require a comparison of the network structure of a set of genes between varying phenotypes. In principle, separately fit models can be directly compared, but it is difficult to assign statistical significance to any observed differences. There would therefore be an advantage to the development of a rigorous hypothesis test for homogeneity of network structure. In this paper, a generalized likelihood ratio test based on Bayesian network models is developed, with significance level estimated using permutation replications. In order to be computationally feasible, a number of algorithms are introduced. First, a method for approximating multivariate distributions due to Chow and Liu (1968) is adapted, permitting the polynomial-time calculation of a maximum likelihood Bayesian network with maximum indegree of one. Second, sequential testing principles are applied to the permutation test, allowing significant reduction of computation time while preserving reported error rates used in multiple testing. The method is applied to gene-set analysis, using two sets of experimental data, and some advantage to a pathway modelling approach to this problem is reported.

## 1. Introduction

Graphical models play a central role in modelling genomic data, largely because the pathway structure governing the interactions of cellular components induces statistical dependence naturally described by directed or undirected graphs [1–3]. These models vary in their formal structure. While a *Boolean network* can be interpreted as a set of state transition rules, *Bayesian* or *Markov networks* reduce to static multivariate densities on random vectors extracted from genomic data. Such densities are designed to model coexpression patterns resulting from functional cooperation. Our concern will be with this type of multivariate model. Although the ideas presented here extend naturally to various forms of genomic data, to fix ideas we will refer specifically to multivariate samples of microarray gene expression data.

In this paper, we consider the problem of comparing network models for a common set of genes under varying phenotypes. In principle, separately fit models can be directly compared. This approach is discussed in [3] and is based on distances definable on a space of graphs. Significance levels are estimated using replications of random graphs similar in structure to the estimated models.

The algorithm proposed below differs significantly from the direct graph approach. We will formulate the problem as a two-sample test in which significance levels are estimated by randomly permuting phenotypes. This requires only the minimal assumption of independence with respect to subjects.

Our strategy will be to confine attention to Bayesian network models (Section 2). Fitting Bayesian networks is computationally difficult, so a simplified model is developed for which a polynomial-time algorithm exists for maximum likelihood calculations. A two-sample hypotheses test based on the general likelihood ratio test statistic is introduced in Section 3. In Section 4, we discuss the application of sequential testing principles to permutation replications. This may be done in a way which permits the reporting of error rates commonly used in multiple testing procedures. In Section 5, the methodology is applied to the problem of *gene set* (GS) analysis, in which high dimensional arrays of gene expression data are screened for *differential expression* (DE) by comparing gene sets defined by known functional relationships, in place of individual gene expressions. This follows the paradigm originally proposed in *gene set enrichment analysis* (GSEA) [4–6]. The method will be applied to two well-known microarray data sets.

An R library of source code implementing the algorithms proposed here may be downloaded at http://www.urmc.rochester.edu/biostat/people/faculty/almudevar.cfm.

## 2. Network Models

A graphical model is developed by defining each of  genes as a graph node, labelled by gene expression level  for gene . The model incorporates two elements, first, a *topology* (a directed or undirected graph on the  nodes), then, a multivariate distribution  for  which conforms to  in some well defined sense. In a *Bayesian network* (BN), model  is a *directed acyclic graph* (DAG), and  assumes the form(1)

where  is the set of parents of node . Intuitively,  describes a causal relationship between node  and nodes .

The advantage of (1) is the reduction in the degrees of freedom of the model while preserving coexpression structure. Also, some flexibility is available with respect to the choice of the conditional densities of (1), with Gaussian, multinomial, and Gamma forms commonly used [7]. We note that BNs are commonly used in many genomic applications [7–9].

### 2.1. Gaussian Bayesian Network Model

For this application, we will use the Gaussian BN. These models are naturally expressed using a linear regression model of node  data  on the data , . In [10], it is noted that in microarray data gene expression levels are aggregated over large numbers of individual cells. Linear correlations are preserved under this process, but other forms of dependence generally will not be, so we can expect linear regression to capture the dominant forms of interaction which are statistically observable. In this case the maximum log-likelihood function for a given topology reduces to(2)

where  is the mean squared error of a linear regression fit of the offspring expressions onto those of the parents.

### 2.2. Restricted Bayesian Networks

Fitting BNs involves optimization over the space of topologies and hence is computationally intensive [9]. While exact algorithms are available [11], they will generally require too great a computation time for the application described below. A recent application of exact techniques to the problem of pedigree reconstruction (a BN with maximum indegree of 2) was described in [12]. Using methods proposed in [13] the exact computation of the maximum likelihood of a pedigree with 29 individuals (nodes) required 8 minutes. The author of [12] agrees with the conclusion reported in [13], that the method is not viable for BNs with greater than 32 nodes.

It is possible to control the size of the computation by placing a cap  on the permissable indegree of each node, though the problem remains difficult even for  (see, e.g., [14]). On the other hand, a method for fitting BNs with constraint  in polynomial time is available under certain assumptions satisfied in our application. This method is based on the equivalence of the approximation of multivariate probability models using tree-structured dependence and the minimum spanning tree (MST) problem as described in [15]. The objective is the minimization of an information difference , where  is the target density, and  is selected from a class of tree-structured approximating densities. Interest in [15] is restricted to discrete densities. We find, however, that the basic idea extends to general BNs in a natural way. See [16] for further discussion of this model.

Many heuristic or approximate methods exist for fitting Bayesian networks. See [17] for a recent survey. Such algorithms are usually based on MCMC techniques or heuristic algorithms such as TABU searches [18]. We note that the proposed hypothesis test will depend on the calculation of a maximum likelihood ratio, hence it is important to have reasonable guarantees that a maximum has been reached. Thus, given the choice between an exact solution of a restricted class of models or an approximate solution of a general class of models, the former seems preferable. Considering also that in the application described below a solution is required for cases number in "10 s or 100 s'' of thousands, a polynomial time exact solution to a restricted class of models appears to be the best choice.

Suppose we are given an -dimensional random vector . We will assume that the density is taken from a parametric family , . We write first- and second-order marginal densities  and , with conditional densities . For convenience, we introduce a dummy vector component , for which . Let  be the set of DAGs on nodes  with maximum indegree 1. This means that a graph  may be written as a mapping . If  has indegree 0 set , otherwise  is the parent node of . We must have  for at least one . For each  let  be the set of parameters admitting the BN decomposition(3)

Now suppose we are given  independent and complete replicates  of . Write components . The log likelihood function becomes, for ,(4)

Suppose we may construct estimators , . We then assume there is some selection rule  for each . This will typically be the exact or approximate maximum likelihood estimate (MLE) on parameter space . We will need the following assumptions. 

  (A1) For each , , and . 

  (A2) For each  we have . 

We now consider the problem of maximizing  over . It will be convenient to isolate the term(5)

A *spanning tree* on nodes  is an acyclic connected undirected graph. Given edge weights , a *minimum spanning tree* (MST) is any spanning tree minimizing the sum of its edge weights among all spanning trees. A number of well-known polynomial time algorithms exist to construct a MST. Two that are commonly described are Prim's and Kruskal's algorithms [19]. Kruskal's algorithm is described in [15]. In the following theorem, the problem of maximizing  is expressed as a MST problem.

Theorem 1.

If assumptions  hold, then maximizing  over  is equivalent to determining the MST for edge weights .

Proof.

Under assumption (A1), from definition (4) it follows that  depends on  only through the term . Then suppose  maximizes . For any spanning tree  define  and suppose  minimizes . Assume  is not connected. There must be at least two nodes  for which , and for which the respective subgraphs containing  are unconnected. In this case, extend  to  by adding directed edge . We must have , and by (A2) we have . We may therefore assume  is connected. The undirected graph of  is a spanning tree, so .

Next, note that  can be identified with an element of  by defining any node as a root node, enumerating all paths from the root node to terminal nodes, then assigning edge directions to conform to these paths. This implies , which in turn implies , and that  may be selected so that  can be identified with .

Remark 1.

In general, the optimizing graph from  will not be unique. First, the solution to the MST problem need not be unique. Second, there will always be at least two extensions of a spanning tree to a BN.

Marginal means, variances and, correlations of  are denoted , leading to parameters , . Each parameter in the set  represents the class of Gaussian BNs which conform to graph . Following the construction in assumption (A1), let ,  using summary statistics , , . Under the usual parameterization, it can be shown that (omitting constants)(6)

noting that, since , assumption (A2) holds.

## 3. General Maximum Likelihood Ratio Test

Identification of nonhomogeneity between two Bayesian networks will be based on a general maximum likelihood ratio test (MLRT). It is important to note the properties of the MLRT are well understood in parametric inference of limited dimension, and a sampling distribution can be accurately approximated with a large enough sample size. These known properties no longer apply in the type of problem considered here, primarily due to the small sample size, large number of parameters, and the fact that optimization over a discrete space is performed. In addition, the maximum likelihood principle itself favors spurious complexity when no model selection principles are used. While we cannot claim that the MLRT possesses any optimum properties in this application, the use of a permutation procedure will permit accurate estimates of the observed significance level while the use of the restricted model class will control to some degree the degrees of freedom of the model. See, for example, [20] for a general discussion of these issues.

Suppose  is a family of densities defined on some parameter set . We are given two random samples  and  from respective densities  and . Denote pooled sample . The density of  and , respectively, are  and . We consider null hypothesis . Under  the joint density of  is  for some parameter . Assume the existence of maximum likelihood estimators , , and . The general likelihood ratio statistic in logarithmic scale is then (with large values rejecting )(7)

Asymptotic distribution theory is not relevant here due to small sample size and the fact that optimization is performed in part over a discrete space of models, so a two sample permutation procedure will be used. Permutations will be approximately balanced to reduce spurious variability when a true difference in expression pattern exists (see, e. g., [21] for discussion). This can be done by changing group labels of  randomly selecting sample vectors from each of  and . This results in permutation replicate samples  and . The balanced procedure ensures that each permutation replicate sample contains approximately equal proportions of the original samples.

We now define Algorithm 1. 

Algorithm 1.

(1) Determine  by maximizing , ,  (MST algorithm).

 () Set .

 () Construct  replications  in the following way. For each replication , create random replicate samples  and , then determine  which maximize , . Set .

 () Set -value (8)

Note that the quantity  is permutation invariant and hence need not be recalculated within the permutation procedure.

## 4. Permutation Tests with Stopping Rules

Permutation or bootstrap tests usually reduce to the estimation of a binomial probability by direct simulation. Since interest is usually in identifying small values, it would seem redundant to continue sampling when, for example, the first ten simulations lead to an estimate of 1/2. This suggests that a stopping rule may be applied to permutation sampling, resulting in significant reduction in computation time, provided it can be incorporated into a valid inference statement. A variety of such procedures have been described in the literature but do not seem to have been widely adopted in genomic discovery applications [22–24].

Suppose, as in Algorithm 1, we have an observed test statistic , and can simulate indefinitely a sequence  from a null distribution . By convention we assume that large values of  tend to reject the null hypothesis. To develop a stopping rule for this sequence set(9)

Formally,  is a *stopping time* if the occurrence of event  can be determined from . We may then design an algorithm which terminates after sampling a sequence of exactly length  from , then outputs , from which the hypothesis decision is resolved. We refer to such a procedure as a *stopped procedure*. A *fixed procedure* (such as Algorithm 1) can be regarded as a special case of a stopped procedure in which .

An important distinction will have to be made between a single test and a *multiple testing procedure* (MTP), which is a collection of  hypothesis tests with rejection rules that control for a global error rate such as *false discovery rate* (FDR), *family-wise error rate* (FWER), or *per family error rate* (PFER) [25]. In the single test application, we may set a fixed significance level  and continue replications until we conclude that the -value is above or below . For an MTP, it will be important to be able to estimate small -values, so a stopping rule which permits this is needed. Although the two cases have different structure, in our development they will both be based on the *sequential probability ratio test* (SPRT), first proposed in [26], which we now describe.

### 4.1. Sequential Probability Ratio Test (SPRT)

Formally (see [27, Chapter 2]) the SPRT tests between two simple alternatives :  versus : , where  parametrizes a family of distributions . We assume there is a sequence of  observations  from  where . Let  be the likelihood function based on  and define the likelihood ratio statistic . For two constants , define stopping time(10)

It can be shown that . If  we conclude  and conclude  otherwise. We define errors  and . It turns out that the SPRT is optimal under the given assumptions in the sense that it minimizes  among all sequential tests (which includes fixed sample tests) with respective error probabilities no larger than . Approximate formulae for  and  are given in [27].

Hypothesis testing usually involves composite hypotheses, with distinct interpretations for the null and alternative hypothesis. One method of adapting the SPRT to this case is to select surrogate simple hypotheses. For example, to test  versus , we could select simple hypotheses  and . In this case, we would need to know the entire power function, which may be estimated using simulations.

An additional issue then arises in that the expected stopping time may be very large for . This can be accommodated using truncation. Suppose a reasonable choice for a fixed sample size is . We would then use truncated stopping time , with  defined in (10). When , we could, for example, select hypothesis  if . These modifications are discussed in [27].

### 4.2. Single Hypothesis Test

Suppose we adopt a fixed significance level  for a single hypothesis test. If  is the (unknown) true significance level, we are interested in resolving the hypothesis :. The properties of the test are summarized in a power curve, that is, the probability of deciding  is true for each . An example of this procedure is given in [28], for , using a SPRT with parameters , , , , and truncation at . Hypothesis  is concluded if  when ; otherwise when .

### 4.3. Multiple Hypothesis Tests

We next assume that we have  hypothesis tests based on sequences of the form (9). We wish to report a global error rate, in which case specific values of small -values are of importance. We will consider specifically the class of MTPs referred to as either *step-up* or *step-down* procedures. If we are given a sequence of -values  which have ranks , then *adjusted**-values*,  are given by:(11)

where the quantity  defines the particular MTP. It is assumed that  is an increasing function of  for all . The procedure is implemented by rejecting all null hypotheses for which . Depending on the MTP, various forms of error, usually either *family-wise error rate* (FWER) or *false discovery rate* (FDR), are controlled at the  level. For example, the Benjamini-Hochberg (BH) procedure is a step-up procedure defined by  and controls for FDR for independent hypothesis tests. A comprehensive treatment of this topic is given in, for example, [25].

Suppose we have  probabilities  (-values associated with  tests). For each test , we may generate  as the cumulative sum defined in (9). Now suppose we define any stopping time , bounded by , for each sequence  (this may or may not be related to the SPRT). Then define estimates , with .

For a fixed MTP, the estimates  would replace the true values in (11), yielding estimated adjusted -values  while for the stopped MTP adjusted -values  are produced in the same manner using . It is easily seen that  while the rankings of  (accounting for ties) are equal to the rankings of . Furthermore, the formulae in (11) are monotone in , so we must have . Thus, the stopped procedure may be seen as being embedded in the fixed procedure. It inherits whatever error control is given for the fixed MTP, with the advantage that the calculation of the adjusted -values  uses only the first  replications for the th test.

The procedure will always be correct in that it is strictly more conservative than the fixed MTP in which it is embedded, no matter which stopping time is used. The remaining issue is the selection of  which will equal  for small enough values of  but will also have  for larger values of . It is a simple matter, then, to modify the SPRT described in Section 4.2 by eliminating the lower bound  (equivalently ). We will adopt this design in this paper. This gives Algorithm 2. 

Algorithm 2.

(1) Same as Algorithm 1, step 1. 

 () Same as Algorithm 1, step 2. 

 () Simulate replicates  in Algorithm 1, step 3, until the following stopping criterion is met. Set , and let , where . Stop sampling at the th replication if , where , or until , whichever occurs first. 

 () Let  be the number of replications in step 3. If , set (12)

otherwise set .

The values  generated by Algorithm 2 can then be used in a stopped MTP as described in this section.

## 5. Gene-Set Analysis

A recent trend in the analysis of microarray data has been to base the discovery of phenotype-induced DE on gene sets rather than individual genes. The reasoning is that if genes in a given set are related by common pathway membership or other transcriptional process, then there should be an aggregate change in gene expression pattern. This should give increased statistical power, as well as enhanced interpretability, especially given the lack of reproducibility in univariate gene discovery due to the stringent requirements imposed by multiple testing adjustments. Thus, the discovery process reduces to a much smaller number of hypothesis tests with more direct biological meaning. Some objections may be raised concerning the selection of the gene sets when theses sets are themselves determined experimentally. Additionally, gene sets may overlap. While these problems need to be addressed, it is also true that such gene set methods have been shown to detect DE not uncovered by univariate screens.

A crucial problem in gene set analysis is the choice of test statistic. The problem of testing against equality of random vectors in , , is fundamentally different from the univariate case . The range of statistics one would consider for  is reasonably limited, the choice being largely driven by distributional considerations. For , new structural or geometric considerations arise. For example, we may have differential expression between some but not all genes in the gene set, which makes selection of a single optimal test statistic impossible. Alternatively, the experimental random vectors may differ in their level of coexpression independently of their level of marginal DE.

In fact, almost all GS procedures directly measure aggregate DE, so an important question is whether or not phenotypic variation is almost completely expressible as DE. If so, then a DE based statistic will have fewer degrees of freedom, hence more power, than one based on a more complex model. Otherwise, a reasonable conjecture is that a compound GS analysis will work best, employing a DE statistic as well as one more sensitive to changes in coexpression patterns.

Correlations have been used in a number of gene discovery applications. They may be used to associate genes of unknown function with known pathways [29, 30]. Additionally, a number of GS procedures exist which incorporate correlation structure into the procedure [31–33]. However, a direct comparison of correlations is not practical due to the large number () of distinct correlation parameters. Therefore, there is a considerable advantage to the statistic (7) based on the reduced BN model, in that the correlation structure can be summarized by the  correlation parameters output by the MST algorithm, yielding a transitive dependence model similar to that effectively exploited in [29].

It is important to refer to a methodological characterization given in [34]. A distinction is made between two types of null hypotheses. Suppose we are given samples of expression levels from a gene set  from two phenotypes. Suppose also that for each gene in  and its complement , a statistical measure of differential expression is available. For a *competitive test*, the null hypothesis  is that the prevalence of differential expression in  is no greater than in . For a *self-contained test*, the null hypothesis  is that no genes in  are differentially expressed. In the GSEA method of [4, 5] concern is with . In most subsequent methods, including the one proposed here,  is used.

For general discussions of the issues raised here, see [35–37]. Comprehensive surveys of specific methods can be found in [38] or [39].

### 5.1. Experimental Data

We will demonstrate the algorithm proposed here on two data sets examined elsewhere in the literature. These were obtained from the GSEA website *www.broad.mit.edu/gsea* [6]. In [5], a data set *p53* is extracted from the NCI-60 collection of cancer cell lines, with 17 cell lines classified as normal, and 33 classified as carrying mutations of p53. We also examine the *DIABETES* data set introduced in [4], consisting of microaray profiles of skeletal muscle biopsies from 43 males. For the *DIABETES* data set used here, there were 17 normal glucose tolerance (*NGT*) subjects and 17 diabetes (*DMT*) subjects. For gene sets, we used one of the gene set lists compiled in [5], denoted , consisting of 472 gene sets with products collectively involved in various metabolic and signalling pathways, as well as 50 sets containing genes exhibiting coregulated response to various perturbations. In our analyses, FDR will be estimated using the BH procedure.

#### 5.1.1. P53 Data

A -test was performed on each of the 10,100 genes. Only 1 gene had an adjusted -value less than FDR = 0.25 (*bax*, , ). Several GS analyses for this data set (using ) have been reported. We cite the GSEA analysis in [5] and a modification of the GSEA proposed in [40]. Also, in [38], this data set is used to test three procedures, each using various standardization procedures. Two are based on logistic regression (*Global test* [41] *ANCOVA Global test* [42]). The third is an extension of the *Significance Analysis of Microarray* (SAM) procedure [43] to gene sets proposed in [44] (SAM-GS).

Table 1 lists pathways selected from  for the analysis proposed here using FDR  0.25, including unadjusted and adjusted -values. For each entry we indicate whether or not the pathway was selected under the analyses reported in [5] (*Sub*, FDR  0.25), [40] (*Efr*, FDR  0.1) and [38] (*Liu*, nominal -value  in at least one procedure). It is important to note that the results indicated with an asterisk (*) are not directly comparable due to differing MTP control, and are included for completeness.

**Table 1:** P53 pathways, with GS size (), unadjusted and FDR adjusted -values ()

Pathway				Sub	Efr	Liu
SA_G1_AND_S_PHASES	14	.001	.08	n	y	n
atmPathway	19	.001	.08	n	n	y
g2Pathway	23	.001	.08	n	n	n
p53Pathway	16	.001	.08	y	y	y
cell_cycle_checkpointII	10	.001	.08	n	n	n
SA_FAS_SIGNALLING	9	.002	.14	n	n*	n*
cellcyclePathway	23	.002	.16	n	n*	n*
DNA_	90	.003	.17	n	n*	n*
SA_TRKA_RECEPTOR	16	.003	.17	n	n*	y*
radiation_sensitivity	26	.003	.17	y	y*	y*
ngfPathway	19	.004	.17	n	y*	n*
GO_ROS	23	.004	.17	n	n*	n*
etsPathway	16	.004	.17	n	n*	n*
ck1Pathway	15	.006	.21	n	n*	n*
erkPathway	29	.007	.23	n	n*	n*
	18	.007	.23	n	n*	n*
arfPathway	13	.007	.23	n	n*	n*

Inclusion in analyses cited in Section 5.1 indicated. The complete name of DNA_DAMAGE is DNA_DAMAGE_SIGNALLING. The complete name of MAP00562 is MAP00562_Inositol_phosphate_metabolism. *Inclusion criterion based on control rate of original analysis.

**Figure 1:**
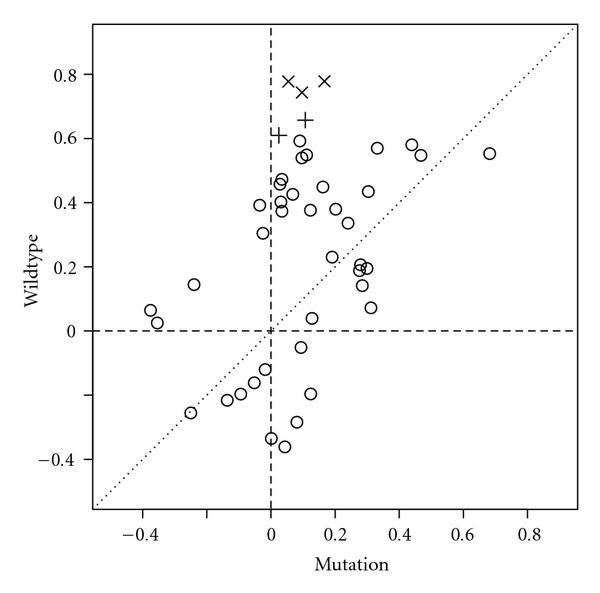
**Scatterplot of correlations for all gene pairs in cell_cycle_checkpoint_II pathway, using wildtype and mutation axes**. Genes with nominal significance levels for differential coexpression  () and  () are indicated separately.

The first five pathways are directly comparable. Of these, two were not detected in any other analysis. Our procedure was repeated for these pathways using the sum of the squared t-statistics across genes. The nominal -values for *g2 Pathway* and *cell cycle checkpoint II* were.0044 and .05, respectively. Since we are interested in identifying pathways which may be detectable by pathway methods, but not DE based methods we will examine *cell cycle checkpoint II* more closely. Applying a univariate -test to each of the 10 genes yields one -value of 0.001 (*cdkn2a*), with the remaining -values greater than 0.1 hence a DE-based approach is unlikely to select this pathway. Furthermore, -values under 0.05 for change in correlation are reported for *rbbp8/rb1*, *nbs1/ccng2*, *atr/ccne2*, *nbs1/tp53, and ccng2/tb53* (,   .006,   .008,   .035,  and  .036). Clearly, the difference in gene expression pattern is determined by change in coexpression pattern. In Figure 1, the correlations for all gene pairs for wild-type and mutation groups are indicated. A clear pattern is evident, by which correlation structure present in the wildtype class does not exist in the mutation class.

**Figure 2:**
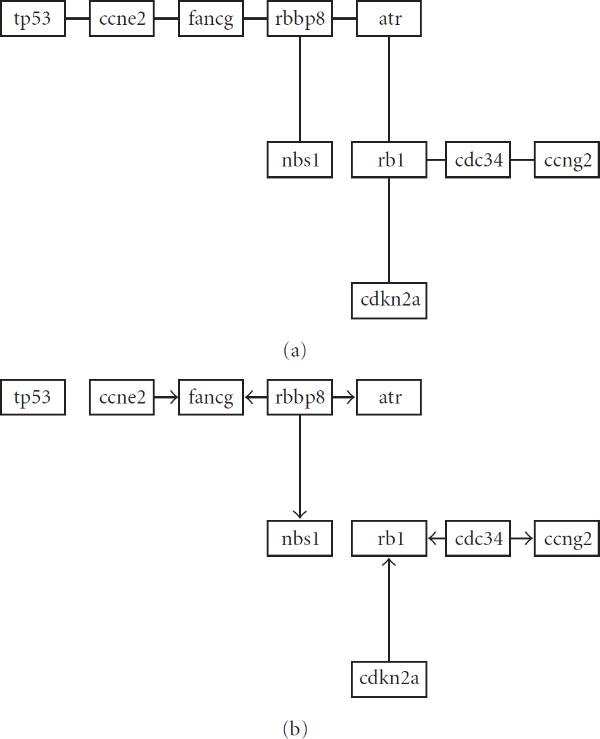
**Bayesian network fits for mutation data for cycle checkpoint II pathway using (a) Minimum Spanning Tree algorithm (maximum indegree of 1); (b) Bayesian Information Criterion (maximum indegree of 2)**.

**Figure 3:**
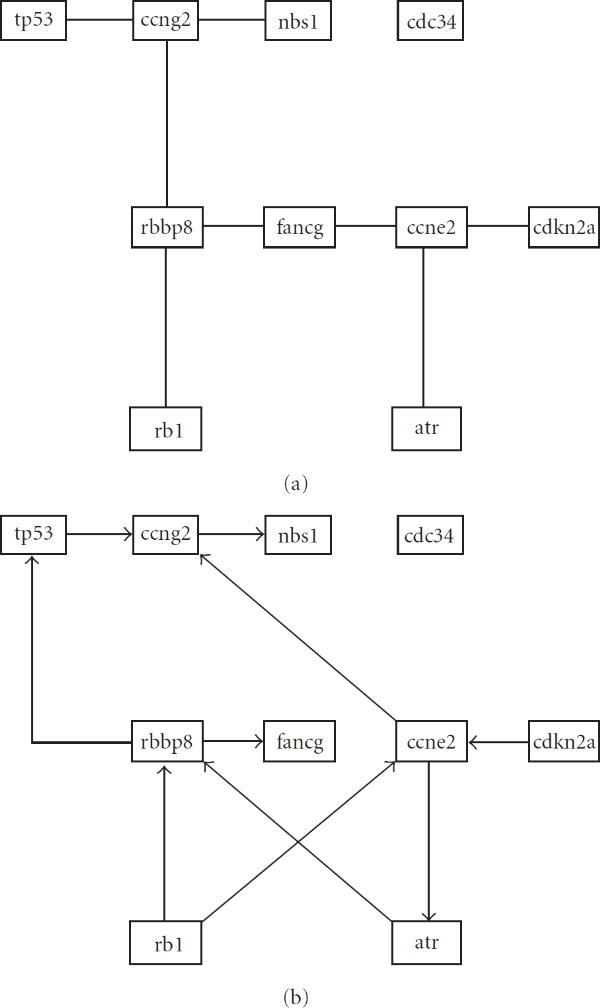
**Bayesian network fits for wildtype data for cycle checkpoint II pathway using (a) Minimum Spanning Tree algorithm (maximum indegree of 1)**. (b) Bayesian Information Criterion (maximum indegree of 2).

To further clarify the procedure, we compare the BN model obtained from the data for the ten genes associated with the *cell cycle checkpoint II* pathway, separately for mutation and wildtype conditions. If there is interest in a post-hoc analysis of any particular pathway, the rational for the MST algorithm no longer holds, since only one fit is required. It is therefore instructive to compare the MST model to a more commonly used method. In this case, we will use the Bayesian Information Criterion (BIC) (see, e.g., [7]), with a maximum indegree of 2. To fit the model we use a simulated annealing algorithm adapted from [45]. The resulting graphs are shown in Figures 2 (mutation) and 3 (wildtype). The MST and BIC fits are labelled (a) and (b) respectively. For the mutation fit, there is a very close correspondence between the topologies produced by the respective methods. For the wildtype data, some correspondence still exists, but less so then for the mutation data. The topologies between the conditions differ more significantly, as predicted by the hypothesis test.

#### 5.1.2. Diabetes

No pathways were detected at a FDR of 0.25. The two pathways with the smallest -values were *atrbrca Pathway* and *MAP00252 Alanine and aspartate metabolism* (). In [33] the latter pathway was the single pathway reported with PFER = 1. The comparable PFER rate of the two pathways reported here would be 1.36 and 1.57. The *atrbrca Pathway* contains 25 genes. Of these, only *fance* differentially expressed at a 0.05 significance level (). For each gene pair, correlation coefficients were calculated and tested for equality between classes *NGT* and *DMT*. Table 2 lists the 10 highest ranking gene pairs in terms of correlation magnitude within the *NGT* class. Also listed is the corresponding correlation within the *DMT* class, as well as the two-sample -value for correlation difference. The analysis is repeated after exchanging classes, also in Table 2. We note that for a sample size of 17, an approximate 95% confidence interval for a reported correlation of  is  whereas the standard deviation of a sample correlation coefficient of mean zero is approximately 0.27. There is likely to be considerable statistical variation in graphical structure under the null hypothesis.

**Table 2:** Correlation analysis for *DIABETES* data

atr brca pathway				Alanine pathway			
***NGT***	**cor**			***NGT***	**cor**		
**genes**	***ngt***	***dmt***	****	**genes**	***ngt***	***dmt***	****

fancc/rad17	83	69	349	crat/got1	81	30	031
fancc/brca2	76	44	156	nars/dars	80	24	1
rad9a/rad17	76	87	338	crat/gpt	75	15	028
chek2/rad17	71	35	172	got2/adss	75	02	012
brca1/hus1	69	29	148	got2/abat	73	34	001
rad17/brca2	67	56	632	ddx3x/got1	72	17	004
atr/mre11a	64	41	403	crat/ass	72	12	037
chek1/nbs1	62	09	030	ddx3x/dars	71	12	043
rad51/rad1	62	23	198	gpt/got1	70	33	175
rad9a/fancc	59	76	388	ddx3x/abat	68	41	305

*DMT*	cor			*DMT*	cor		
genes	*dmt*	*ngt*		genes	*dmt*	*ngt*	

rad9a/rad17	87	76	338	ddx3x/aars	76	55	325
fanca/fance	81	14	009	crat/nars	74	26	074
rad9a/fancc	76	59	388	ddx3x/nars	73	66	715
fanca/hus1	72	27	002	asns/ddo	60	42	502
brca1/mre11a	71	11	039	pc/aars	58	15	031
fancc/rad17	69	83	349	crat/pc	58	53	862
fancf/hus1	67	53	563	crat/ddx3x	58	51	813
brca1/atr	67	16	011	got1/dars	56	40	006
rad17/mre11a	64	11	086	pc/nars	55	18	244
fancg/rad51	64	22	160	asns/gad2	54	44	723

For each pathway and phenotype, 10 gene pairs with the largest correlation (100) magnitudes; correlation (100) of alternative phenotype; and -value (1000) against equality.

Examining the first table, differences in correlation appear to be explainable by sampling variation. In the second there are two gene pairs *fanca/fance* and *fanca/hus1* with small -values (.009,   .002). We note that they share a common gene *fanca* and that they involve the only gene *fance* exhibiting differential expression. The correlation patterns within the two samples are otherwise similar, suggesting a specific alteration of the network model.

The situation differs for the pathway *MAP00252 Alanine and aspartate metabolism*, summarized in Table 2 using the same analysis. The change in correlation is more widespread. The 8 gene pairs with the highest correlation magnitudes within the *NGT* sample differ between *NGT* and *DMT* at a 0.05 significance level. Furthermore, the number of gene pairs with correlation magnitudes exceeding 0.7 is 9 in the *NGT* sample, but only 3 in the *DMT* sample.

#### 5.1.3. Comparison of Fixed and Stopped Procedures

Both the fixed and stopped procedures were applied to the preceding analysis. The SPRT used parameters , , , , and truncation at . Table 3 summarizes the computation times for each method as well as the selection agreement. In these examples, the stopped procedure required significantly less computation time with no apparent loss in power.

**Table 3:** For stopped (*St*) and fixed (*Fx*) procedures, the table gives computation times; mean number of replications; % gene sets completely sampled; number of pathways with -values ; 01; and number of such pathways in agreement.

Data	Time (*hrs*)		Mean rep		% comp		^#^		
	*St*	*Fx*	*St*	*Fx*	*St*	*Fx*	*St*	*Fx*	Both
*diab*	3.7	35.8	341.0	5000	5.4	100	6	6	6
*p53*	2.1	30.0	612.3	5000	10.5	100	18	19	18

## 6. Conclusion

We have introduced a two-sample general likelihood ratio test for the equality of Bayesian network models. Significance levels are estimated using a permutation procedure. The algorithm was proposed as an alternative form of gene-set analysis. It was noted that the fitting of Bayesian networks is computationally time consuming, hence a need for the efficient calculation of a model fit was identified, particularly for this application.

Two procedures were introduced to meet this requirement. First, we implemented a version of a minimum spanning tree algorithm first proposed in [15] which permits the polynomial-time calculation of the maximum likelihood Bayesian network among those with maximum indegree of one. Second, we introduced sequential testing principles to the problem of multiple testing, finding that a straightforward stopping rule could be developed which preserves group error rates for a wide range of procedures.

We may expect this form of test to be especially sensitive to changes in coexpression patterns, in contrast to most gene-set procedures, which directly measure aggregate differential expression. In an application of the algorithm to two data sets considered in [5], a number of selected gene-sets exhibited clear differences in coexpression patterns while exhibiting very little differential expression. This leads to the conjecture that the optimal approach to gene-set analysis is to couple a test which directly measures aggregate differential expression with one designed to detect differential coexpression.
